# Extraction of a Triterpene Solution and Evaluation of the Hypolipidemic Efficacy of the *Pleurotus tuber-regium* (Fr.) Sing Sclerotium

**DOI:** 10.3390/foods11182881

**Published:** 2022-09-16

**Authors:** Chao Wang, Yuan Liu, Yuanhong Lan, Jianing Yuan

**Affiliations:** College of Food and Health, Zhejiang A & F University, Hangzhou 311300, China

**Keywords:** *Pleurotus tuber-regium* (Fr.) Sing Sclerotium, total triterpenes, hypolipidemic

## Abstract

The total triterpenes in edible mushrooms have high medicinal value, and the sclerotium has various biological activities, such as the regulation of blood pressure and blood glucose. In this study, the total triterpenes of the *Pleurotus tuber-regium* (Fr.) Sing Sclerotium (PTRSS) were extracted, and their hypolipidemic effects were also investigated. The infrared spectra showed that the total triterpenes were consistent with the characteristic structures of the total triterpenes before and after purification. The binding abilities of total triterpenes to sodium glycocholate, sodium taurocholate, and sodium cholate were investigated, and all of them had a good binding ability to cholate. In vivo experiments showed that zebrafish tolerated the total triterpenes from the mushroom nuclei at a maximum concentration of 500 µg/mL. A correlation analysis showed that the total triterpenes from the mushroom nuclei reduced the lipid accumulation in zebrafish induced by a high-fat diet, and the lipid-lowering effect showed a correlation with dose.

## 1. Introduction

Hyperlipidemia is a disorder of the lipid metabolism, and chronic hyperlipidemia can lead to a range of diseases, such as atherosclerosis, stroke, coronary heart disease, myocardial infarction, diabetes, and kidney failure [1,2,3]. Drug treatments for hyperlipidemia can bring about a number of adverse effects, so lipid-lowering active substances have drawn great attention because they are natural, have a low toxicity, and are effective [4,5]. At present, the effective blood-lipid-lowering drugs that are clinically used include fibrates, statins, etc. Although they have a strong regulatory effect on blood lipids, Western medicines usually have a high toxicity and side effects, and long-term use can easily induce other diseases, which is not good for human health. The breadth and complexity of the living environments of edible and medicinal fungi make it possible to discover more valuable active substances compared with those found in other organisms.

The total triterpenes in medicinal mushrooms have hypolipidemic, hypoglycemic, anti-fatigue, and diuretic effects [6,7]. Compared with other active substances (e.g., polysaccharides, flavonoids), they have a high medicinal value and have been applied to the clinical treatment of some diseases [8]. In recent years, terpenoids, including sesquiterpenes, diterpenes, and triterpenes, have been isolated from edible medicinal mushrooms [9,10,11]. Previous studies showed that total triterpenes have the ability to bind cholate in vitro, so the hypolipidemic efficacy of triterpene substances was further explored [12,13]. It has been documented that α,β-amyrin (from a mixture of Protium heptaphyllum triterpenes) has anti-hyperglycemic and lipid-lowering effects [14]. In addition, it may improve glucose tolerance in mice through its anti-inflammatory and antioxidant effects [15]. Some researchers also found the anti-atherosclerotic activity of Chios Mastic gum (in which the main compound is triterpene) when it was evaluated in vivo. The results showed a significant hypolipidemic effect in rabbits that were fed cholesterol-rich foods.

Mycorrhiza are dense and hard tissue structures formed through the asexual reproduction of fungal hyphae under adverse environmental conditions; they provide nutritional storage and are a dormant organ of fungi [16]. The sclerotia of macrofungi are rich in nutrients such as carbohydrates, proteins, polyphosphates, lipids, and minerals, as well as terpenoids, phenols, melanin, and many other bioactive components, which have various biological activities, such as the enhancement of immune function, improvement of intestinal function, antioxidant activity, and the regulation of blood pressure and blood sugar [17,18].

**Scheme 1:**
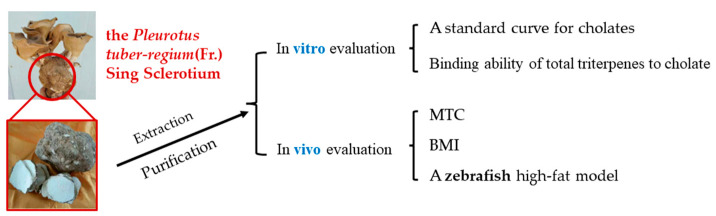
Chart of the overall idea of the research on PTRSS.

In this study, the edible and medicinal fungus of *Pleurotus tuber-regium* (Fr.) Sing Sclerotium (PTRSS) was used as the research object, and the HMGR inhibitor was obtained through modern extraction, separation analysis, and other testing methods; then, the mechanism and efficacy of its inhibition of cholesterol synthesis were explored (Scheme 1). Zebrafish were used as test animals to establish a high-fat model, and the in vivo hypolipidemic activity of total triterpenes from tiger mushroom kernels was analyzed through the lipid accumulation in zebrafish in order to provide some reference for the development of natural and effective hypolipidemic products, as well as to provide an experimental basis for the study of the efficacy of total triterpenes, which would provide a theoretical basis for the development of natural and effective hypolipidemic drugs.

## 2. Materials and Methods

### 2.1. Reagents and Apparatuses

The PTRSS was provided by Linchuan Jinshan Biotechnology Co., Ltd. (Fuzhou, China). Total cholesterol, triglycerides, LDL cholesterol, HDL cholesterol, phosphorothioic iron chromogenic, 85% concentrated phosphoric acid, sodium glycocholate, sodium taurocholate, sodium cholate, trypsin, oleuropein O, and pepsin were provided by Shanghai Yuanye Biotechnology Co., Ltd. (Shanghai, China). Melanin-allele-mutant translucent ab-strain zebrafish and ms-222 anesthetic were provided by Shanghai Fei Xi Biotechnology Co., Ltd. (Shanghai, China). Egg yolk powder and instant sea salt for birds were provided by Beijing Kaiyuan Feed Co., Ltd. (Beijing, China). Kauai Enamine San was provided by Nanjing Hou Sheng Pharmaceutical Co., Ltd. (Nanjing, China). The rest of the reagents were analytically pure. The PTRSS was characterized with a Nicolet 6700 FTIR Spectrometer (Thermo Fisher Scientific, Massachusetts, USA) and centrifuged with an SC-04 centrifuge (Anhui Zhongke Zhongjia Scientific Instrument Co., Ltd., China). Zebrafish were observed with a Zeiss 508 stereomicroscope (Zeiss, Oberkochen, Germany).

### 2.2. Extraction of the Triterpene Solution

The PTRSS was treated according to a previously reported method [19,20]. In brief, the PTRSS was sliced and dried at 60 °C to a constant weight (<10%). The powder was sieved through a 40 mesh and stored in a glass jar. Subsequently, an appropriate amount of the PTRSS was weighed and dissolved in 75% ethanol, shaken well, and then subjected to ultrasonically assisted extraction at 55 °C for 45 min with a fixed extraction power of 300 W and a frequency of 40 KHz. Finally, the mixture was centrifuged at 4000× *g* for 18 min, and the supernatant was collected in a rotary evaporator to evaporate the solvent in order to obtain the total-triterpene-rich extract of the PTRSS.

The purified product was obtained through a wet resin process, as described in a previous study [21,22]. In detail, the D-101-type microporous resin was selected for pretreatment, and the resin was washed with water before being soaked in ethanol to loosen and spread the resin layer. After soaking in ethanol overnight, the resin was extracted and eluted with ethanol until the eluate did not produce turbidity when added to 5 times water. Then, it was eluted with distilled water until no there was alcoholic smell, soaked with 2% hydrochloric acid and 2% sodium hydroxide in turn for 3 h, washed with distilled water until it was neutral, and dried for further use.

The total triterpenes were extracted from the column through the wet loading of resin, the pH was adjusted to 6, and the sample was loaded at a flow rate of 3 BV/h. The eluate was collected and the solvent was evaporated through spinning to obtain the purified total triterpenes from the PTRSS.

### 2.3. Establishment of a Standard Curve for Cholates

First, 2 mL of different concentrations of sodium glycocholate (0.2, 0.4, 0.6, 0.8, and 1.0 µmol/mL) was dissolved in 5 mL of concentrated sulfuric acid (60 wt%) solution. Then, the reaction was carried out in a constant-temperature water bath at 70 °C for 20 min and cooled with ice water for 5 min. Finally, the absorbance values were measured at 387 nm. The standard curve was established with the content of sodium glycocholate as the horizontal coordinate and the absorbance value as the vertical coordinate, and the linear equation of the standard curve was obtained as follows: y = 1.312x + 0.1244, R^2^ = 0.9969. The standard curves for sodium taurocholate and sodium cholate were constructed as described above, and the standard curve equations were y = 2.677x − 0.0702, R^2^ = 0.9954 and y = 4.013x + 0.1287, R^2^ = 0.9984, respectively.

### 2.4. Binding Ability of Total Triterpenes to Cholate

First, different mass concentrations of total triterpenes of tiger mushroom nuclei were taken in conical flasks. After that, pepsin (10 mg/mL) and hydrochloric acid (10 µmol/mL) were added sequentially and incubated at 37 °C for 1 h. Then, the pH was adjusted to 6.4 with NaOH (10 µmol/mL) and trypsin (10 mg/mL) was added, followed by incubation at 37 °C for 1 h. Finally, the supernatant was centrifuged and the absorbance was measured to calculate the amount of cholate binding. Meanwhile, the ability of cholestyramine to bind cholate was determined as described above.

### 2.5. Zebrafish Breeding

A zebrafish animal model was selected for the evaluation of the efficacy, and a high-fat model was established to analyze the in vivo hypolipidemic activity of total triterpenes from the PTRSS by assessing the lipid accumulation in zebrafish [23,24].

Juvenile zebrafish were reared in water after 48 h of aeration under the following experimental conditions: (i) water temperature of 28 °C, pH controlled between 6.9 and 7.2, conductivity of 480–510 µs/cm, and hardness of 53.7–71.6 µg/mL CaCO_3_; (ii) 200 mg of instant sea salt was added per 1 L of reverse-osmosis water, and the fish-rearing water was changed daily; (iii) daylight irradiation for 14 h during the day and placement in the dark for 10 h to simulate the external environment. It is worth noting that the zebrafish fry that were just 9 dpf (9 days post-fertilization) could obtain nutrients from their own vitellogenin and, therefore, did not require additional feeding [25].

### 2.6. Measurement of Maximum Tolerated Concentration (MTC) and Body Mass Index (BMI)

It is well known that the uptake of target substances by juvenile zebrafish is exposure-based. The juvenile zebrafish were exposed to a flat dish containing the target substance, and they absorbed the substance through their own skin and lumen.

Ninety well-developed zebrafish that were 5 dpf were selected and placed in flat dishes that were 10 cm in diameter, and 15 zebrafish were placed in each flat dish. Subsequently, 100 mL of the total triterpene solution with different concentration gradients (100, 300, 500, 700, and 900 µg/mL) was added, and another group was given water for rearing as a blank control group. The zebrafish were kept in the above environment for 48 h, and the physical changes and mortality of the juvenile zebrafish were observed and recorded every 2 h.

The juvenile zebrafish were anesthetized with a 1:10,000 ratio of ms-222 solution. Then, the juvenile zebrafish were moved to a slide, and the water on the surface was blotted with absorbent paper. The BMI of the zebrafish was calculated by weighing each group of zebrafish with an analytical balance and measuring their body length with vernier calipers. The BMI was calculated as shown in Equation (1).

(1) $$\mathrm{BMI}=\frac{Q}{L}$$

where Q and L denote the weight and body length of the zebrafish, respectively.

### 2.7. Construction of a High-Fat Zebrafish Model

Sixty zebrafish at 5 dpf were randomly selected and fed with 1% egg yolk powder solution in a flat dish for 12 h per day for 2 days. At the same time, the zebrafish were stained and photographed after the feeding was completed, and the water was replaced with clean water.

After the high-fat model was established, the zebrafish were randomly divided into four groups of 15 fish each, namely, a high-fat group and high-fat triterpene groups with different concentration gradients (the concentration gradients were selected based on the results of the MTC) [26]. After 2 days, the zebrafish in the high-fat triterpene group were stained and photographed to observe the lipid accumulation in the juvenile zebrafish, and the total lipid optical density was calculated.

Lipid staining in zebrafish: The 5% Oil Red O staining solution was first prepared with isopropyl alcohol, fully dissolved, and then filtered 3 times in a dark room using qualitative filter paper; then, the staining solution was stored in a brown bottle at 4 °C for further use.

For zebrafish lipid staining [27], the zebrafish were first placed in ms-222 anesthetic solution at a concentration of 1:10,000 for 15 min. Subsequently, the anesthetized fish were fixed in 4% formaldehyde solution for 3 h and washed with PBS. Then, the zebrafish were dehydrated with methanol solution from a low concentration to a high concentration. After dehydration, the zebrafish were incubated overnight in the Oil Red staining solution and protected from light. Moreover, the zebrafish were rehydrated with methanol solution from a high to a low concentration and washed with PBS. Finally, they were stored in glycerol and photographed with a stereomicroscope.

Lipid optical density [28,29]: The images were decoded using the Image-J software in the microscope format taken by Zeiss; then, they were processed and analyzed with the Image-Pro Plus (IPP) software to determine the total integrated optical density (IOD) and quantify the average integrated optical density (AIOD) in the zebrafish. The lipid accumulation was calculated with the following equation: Lipid-lowering effect = (AIOD high-fat group−AIOD high-fat triterpene group)/AIOD high-fat group.

### 2.8. Statistical Analysis Methods

The SPSS software was used for the statistical analysis of the experimental data, which were expressed as the mean ± standard deviation (x ± s), and the differences between groups were tested with a *t*-test.

## 3. Results

### 3.1. Analysis of Infrared Spectrograms

**Figure 1:**
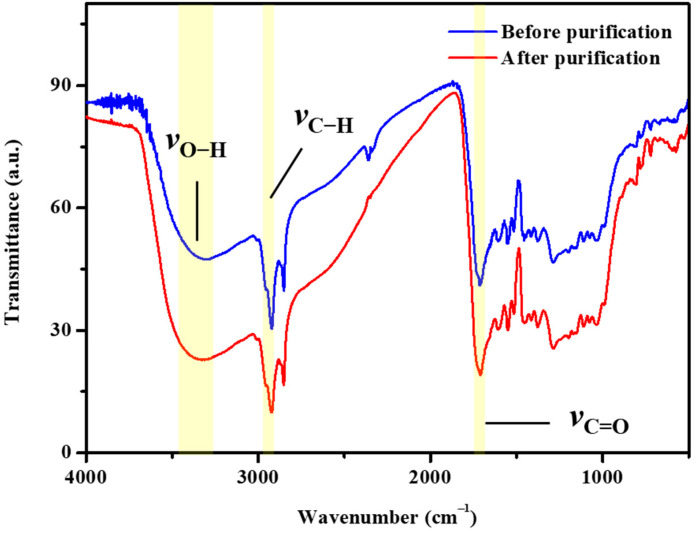
Infrared spectrum of total triterpenoids from the sclerotia of PTRSS.

It can be seen that the total triterpenes of the PTRSS before and after purification had -OH bond stretching vibration peaks at 3328 and 3307 cm^−1^, respectively (Figure 1) [30]. However, the peaks and the peak widths were different. This may have been due to the presence of hydrogen bonds, which changed the electron density distribution and, thus, the stretching vibration frequency. The absorption peak of the total triterpenes was present at 2922 cm^−1^, which was due to the stretching vibration of symmetric or asymmetric -CH_2_ and -CH_3_. The presence of the stretching vibrational peaks of carboxyl C=O bonds at 1713 and 1709 cm^−1^ for the total triterpenes before and after purification, respectively, suggests that the total triterpenes of the PTRSS may be acidic saponins [31]. The total triterpenes before and after purification had only one absorption peak between 1355–1392 and 1245–1330 cm^−1^, suggesting that they belong to tetracyclic triterpenes. In addition, the waveforms of the IR spectra before and after purification were basically similar, and the differences in the characteristic peaks were small; all of them were consistent with the characteristic structures of the total triterpenoids [32]. Therefore, the purified total triterpenes could be subjected to further activity studies.

### 3.2. Analysis of the In Vitro Evaluation

**Figure 2:**
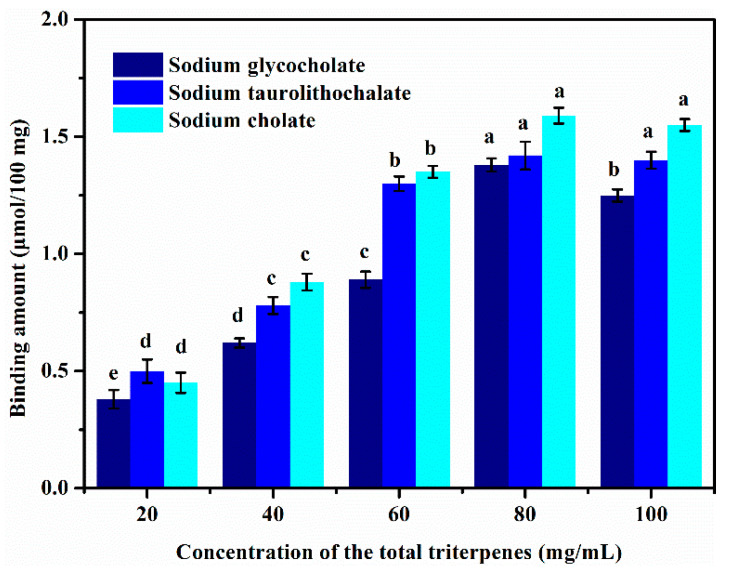
The amount of total triterpenoids bound to cholic acid salt in the sclerotia of PTRSS. Note: There are no significant differences between means with identical letters in a column (*p* < 0.05).

Binding amount of cholate: The binding ability of the total triterpenes of the PTRSS with cholate is shown in Figure 2; the mass concentration of the total triterpenes was positively correlated with the binding amount of cholate in a certain range. When the mass concentration was 20–60 mg/mL, the amounts of total triterpenes that bound to sodium glycocholate, sodium taurocholate, and sodium cholate were significantly increased (*p* < 0.05); when the mass concentration was 80–100 mg/mL, the binding of total triterpenes to cholate was close to saturation. Its binding to sodium taurocholate and sodium cholate did not change significantly (*p* > 0.05), and its binding to sodium glycocholate decreased significantly (*p* < 0.05). Meanwhile, the maximum binding to cholate was observed at a mass concentration of 80 mg/mL.

In vitro hypolipidemic activity: Cholestyramine is a common hypolipidemic drug that mainly acts on cholic acid or cholesterol in the body, and it can effectively improve the blood lipid levels of hyperlipidemia patients with good clinical efficacy. The ability of cholestyramine to bind cholate at 80 mg/mL was used as a control to determine the amount of total triterpenes of the PTRSS bound to cholate at the same mass concentration.

The binding abilities of the total triterpenes to sodium glycocholate, sodium taurocholate, and sodium cholate were equivalent to 50.93%, 52.14%, and 43.06% of the same dose of caustic amine, respectively, indicating that the total triterpenes of the PTRSS had a good ability to bind cholate (Table 1). There are two main methods of lowering cholesterol. (i) Reducing the synthesis of cholesterol in the body: Active substances, such as polysaccharides, are inhibitors of this substance. Many polysaccharides of edible and medicinal fungi can inhibit the biosynthesis of cholesterol in the body by inhibiting its activity or reducing their content, thereby achieving the effect of lowering blood lipids. (ii) Reducing the absorption of exogenous cholesterol: Some biologically active substances can reduce the absorption of cholesterol in the intestine, thereby promoting the excretion of cholesterol with feces from the intestine.

Some researchers have proposed that cholate becomes less abundant after being bound, which will promote the degradation of cholesterol in the liver to produce cholate and maintain the dynamic balance, thus achieving the purpose of lowering blood lipids [33,34,35]. Therefore, it was shown that the total triterpenes of the PTRSS had good in vitro hypolipidemic activity.

### 3.3. Analysis of the In Vivo Evaluation

MTC: As shown in Table 2, the zebrafish showed no significant changes in body condition and 0% mortality at concentrations of 100–500 µg/mL of the total triterpenes of the PTRSS; at a concentration of 700 µg/mL, the mortality rate of the juvenile zebrafish was 6.67%, but this death of only one fish cannot yet allow the exclusion of the possibility of the influence of other factors. At a total triterpene concentration of 900 µg/mL, the mortality rate of the zebrafish larvae was 40% (i.e., 6 out of 15 zebrafish died), and some of the dead zebrafish showed bent bodies. On average, the maximum tolerated concentration of total triterpenes in the juvenile zebrafish was 500 µg/mL.

BMI: Both the body weights and lengths of the juvenile zebrafish in the high-fat group were significantly increased compared to those of the blank group (*p* < 0.05), which related to the susceptibility of people consuming high-fat foods for a long period of time to obesity and other problems (Table 3). However, both the body weights and lengths of the juvenile fish were significantly lower compared to those of the high-fat group after being fed the total triterpenes. In addition, the BMI of the zebrafish was significantly lower compared to that of the high-fat group when the total triterpene concentration was 500 µg/mL, suggesting that the total triterpenes could slow down the changes in the body mass index brought about by a high-fat diet.

**Figure 3:**
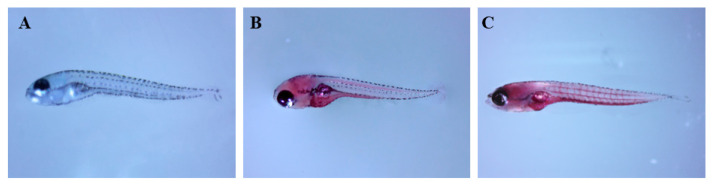
The results of Oil Red O staining after modeling. Note: The zebrafish in the blank control group (**A**) and in the high-fat group were stained for 3 h (**B**) and overnight (**C**).

High-fat zebrafish modeling: The accumulation of lipids induced in the zebrafish by a high-fat diet rose rapidly, and the lipids in their bodies appeared in red after oil red staining. As shown in Figure 3, the zebrafish in the blank control group were transparent all over (Figure 3A), while the zebrafish in the high-fat group and the lipids in their swim bladder were stained red with ovalbumin after 3 h of Oil Red staining, but the lipids in the blood vessels were not stained sufficiently (Figure 3B). This was because there was a correlation between the staining effect and the staining time. When the zebrafish in the high-lipid group were stained overnight, the lipids in both the brain and the blood vessels were fully stained (Figure 3C). However, the high-fat model used in the experimental research process was induced by exogenous food, so it could be speculated that the total triterpenes interfered with the process of blood lipid metabolism by affecting the absorption of exogenous lipids.

Hypolipidemia: All other things being equal, the feedback of different objects at the same light intensity is different (i.e., the transmitted light index is different), and this value can be calculated from the grayscale value. The darker the target substance is, the less light will be transmitted, representing a higher optical density index. Therefore, the optical density value can be used to measure the content of a component in a tissue or cell.

Table 4 and Figure S1 show the lipid staining in the zebrafish and the corresponding lipid optical density results, respectively, and it is evident that the addition of the total triterpenes to the fish culture water significantly reduced the lipid accumulation in the zebrafish. There were no significant changes (*p* > 0.05) in the lipid-lowering effect on zebrafish when the total triterpene concentration was 100 or 300 µg/mL. However, there was a significant lipid-lowering effect (*p* < 0.05) of up to 32.96 ± 3.29% at a total triterpene concentration of 500 µg/mL. These results showed that the total triterpenes of the PTRSS could reduce the lipid accumulation induced in zebrafish by a high-fat diet. Moreover, in the mass concentration range of 100–500 µg/mL of total triterpenes, the higher the mass concentration is, the more significant the effect on lipid reduction will be.

## 4. Conclusions

In summary, the total triterpenes of the PTRSS have a good in vitro hypolipidemic activity. Within a certain range, the greater the mass concentration of total triterpenes is, the stronger their ability to bind cholate will be. The binding abilities of total triterpenes to sodium glycocholate, sodium taurocholate, and sodium cholate were equivalent to 50.93%, 52.14%, and 43.06% of the same dose of cholestyramine, respectively. The in vitro experiments indicated that the total triterpenes from tiger mushroom nuclei could bind cholate better. The in vivo tests showed that the maximum tolerated concentration of the total triterpenes of the PTRSS was 500 µg/mL; the length and body weight of zebrafish in the high-fat model were significantly increased, and the addition of the total triterpenes of the PTRSS could improve this situation properly. In addition, the total triterpenes of the PTRSS reduced the lipid accumulation induced in the zebrafish by a high-fat diet by up to 32.96 ± 3.29% at 500 µg/mL. Although a higher rate of extraction of total triterpenes from PRTSS was obtained and the existence of total triterpenes was confirmed through infrared spectroscopy, the purity and the structure–activity relationship still need to be further explored. In addition, if imaging technology can be used to observe the living state and subsequent morphological changes of zebrafish during the entire experiment, the research will be more comprehensive and vivid.

## Figures and Tables

**Table 1:** The capacities of total triterpenoids from the sclerotia of PTRSS for binding with bile salts in vitro.

	Sodium Glycocholate (μmol/100 mg)	Binding Rate (%)	Sodium Taurocholate (μmol/100 mg)	Binding Rate (%)	Sodium Cholate (μmol/100 mg)	Binding Rate (%)
PTRSS	1.39 ± 0.07	50.93 ± 3.34	1.42 ± 0.07	52.14 ± 4.07	1.68 ± 0.03	43.06 ± 1.71
Cholestyramine	2.73 ± 0.04	100.00 ± 1.57	2.73 ± 0.09	100.00 ± 3.11	3.89 ± 0.08	100.00 ± 2.02

**Table 2:** The results for the MTC.

Group	Concentration of the Total Triterpenoids (µg/mL)	Number of Deaths	Mortality (%)	Body Changes
Blank	-	0	0	-
Triterpenes	100.00	0	0	-
	300.00	0	0	-
	500.00	0	0	-
	700.00	1	6.67	-
	900.00	6	40.00	Bend

**Table 3:** The results for the BMI.

Group	Concentration of the Total Triterpenoids (µg/mL)	Body Weight (mg)	Body Length (mm)	BMI (mg/mm^2^)
Blank	-	20.29 ± 0.73 ^d^	10.97 ± 0.68 ^b^	0.17 ± 0.02 ^b^
High-fat	-	22.65 ± 0.65 ^a^	11.23 ± 0.54 ^a^	0.18 ± 0.02 ^a^
High-fat triterpene	100.00	21.17 ± 0.73 ^b^	10.85 ± 0.31 ^c^	0.18 ± 0.01 ^a^
	300.00	20.73 ± 0.47 ^c^	10.71 ± 0.25 ^c^	0.18 ± 0.01 ^a^
	500.00	20.36 ± 0.72 ^d^	10.84 ± 0.38 ^c^	0.17 ± 0.02 ^b^

The values are expressed as the mean ± standard deviation of three replicates. There are no significant differences between means with identical letters in a column (*p* < 0.05).

**Table 4:** The analysis of the optical density in the zebrafish lipid staining.

Group	Concentration of the Total Triterpenoids (µg/mL)	Area	Integrated Optical Density (IOD)	Average Integrated Optical Density (AIOD)	Lipid-Lowering Effect (%)
High-fat	0	159,371 ± 261.34	1053.34 ± 23.68	0.0066 ± 0.00014 ^a^	-
High-fat triterpene	100.00	134,051 ± 458.87	783.81 ± 34.89	0.0058 ± 0.00019 ^b^	11.54 ± 1.05 ^a^
	300.00	126,218 ± 404.06	683.51 ± 33.17	0.0054 ± 0.00027 ^b^	19.27 ± 4.16 ^a^
	500.00	124,247 ± 539.63	573.59 ± 32.93	0.0046 ± 0.00028 ^c^	32.96 ± 3.29 ^b^

The values are expressed as the mean ± standard deviation of three replicates. There are no significant differences between means with identical letters in a column (*p* < 0.05).

## Data Availability

Data is contained within the article or Supplementary Materials.

## Supplementary Materials

The following supporting information can be downloaded at: https://www.mdpi.com/article/10.3390/foods11182881/s1, Figure S1: The staining results of lipid accumulation in zebrafish. Note: A, B, C and D are zebrafish fed with total triterpenoid concentrations of 0 µg/mL, 100 µg/mL, 300 µg/mL and 500 µg/mL, respectively.
