# Supplementary material for: Extraction of a Triterpene Solution and Evaluation of the Hypolipidemic Efficacy of the Pleurotus tuber-regium (Fr.) Sing Sclerotium
Source: Foods. 2022 Sep 16;11(18):2881. doi: 10.3390/foods11182881 (PMC9498554; doi:10.3390/foods11182881)
Supplement: Supplementary file 1 [file foods-11-02881-s001.zip › foods-1874160-supplementary.pdf]

foods

MDPI

Supplementary Information

Extraction of a Triterpene Solution and Evaluation of the Hypolipidemic Efficacy of the *Pleurotus tuber-regium* (Fr.) Sing Sclerotium

Chao Wang *, Yuan Liu, Yuanhong Lan and Jianing Yuan

College of Food and Health, Zhejiang A & F University, Hangzhou 311300, China
* Correspondence: tianshan@zafu.edu.cn

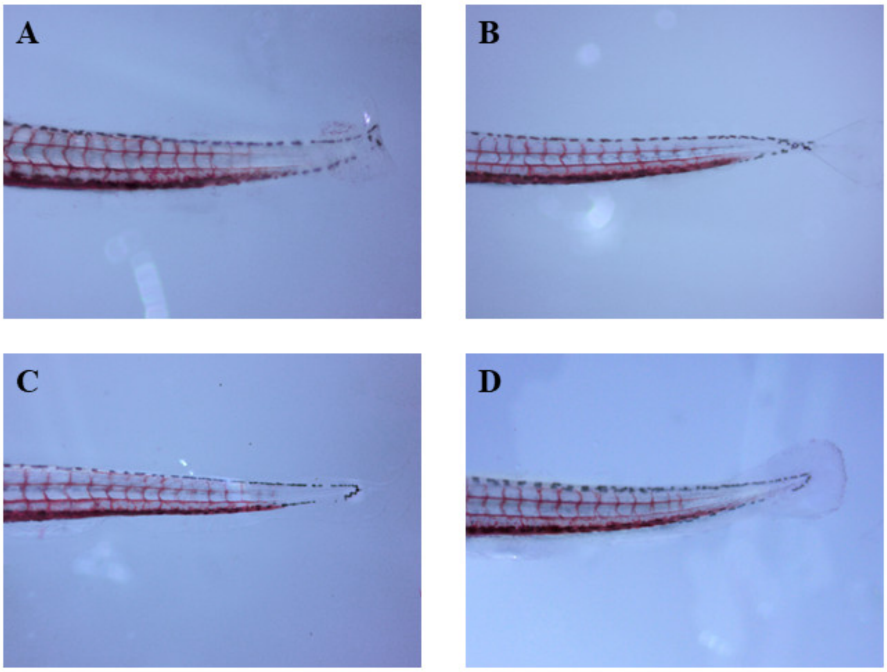

Figure S1. The staining results of lipid accumulation in zebrafish. Note: A, B, C and D are zebrafish fed with total triterpenoid concentrations of 0 μg/mL, 100 μg/mL, 300 μg/mL and 500 μg/mL, respectively.
